# Anatomical outcome after brachytherapy with bi-nuclide (Ru-106/Iodine-125) plaques in large uveal melanomas

**DOI:** 10.1186/s13014-025-02707-7

**Published:** 2025-07-31

**Authors:** Leyla Jabbarli, Miltiadis Fiorentzis, Maja Guberina, Boerge Schmidt, Philipp Rating, Eva Biewald, Nika Guberina, Dirk Flühs, Norbert Bornfeld, Wolfgang Sauerwein, Martin Stuschke, Nikolaos E. Bechrakis

**Affiliations:** 1 Department of Ophthalmology, University Hospital Essen, 45147 Essen, Germany; 2 Department of Radiotherapy, University Hospital Essen, Essen, Germany; 3 German Cancer Consortium (DKTK), Heidelberg, Partner Site University Hospital Essen, Essen, Germany; 4 Institute for Medical Informatics, Biometry and Epidemiology (IMIBE), University of Duisburg-Essen, Essen, Germany

**Keywords:** Uveal melanoma, Brachytherapy, Bi-nuclide plaque, Local recurrence, Enucleation, Risk factors

## Abstract

**Background:**

Proprietary bi-nuclide plaques combine the radiation properties of beta and gamma brachytherapy and can irradiate a larger target volume compared to ruthenium-plaques. While reducing the dose to structures outside the target volume, brachytherapy with bi-nuclide-plaques (BBNP) delivers a higher target dose compared to iodine-plaques. We aimed at analyzing the local tumor control and eye retention probability after BBNP.

**Methods:**

All consecutive cases with large uveal melanoma (tumor thickness ≥ 7 mm) treated with BBNP at our institution between 01/1999 and 12/2020 were included (n = 576, median follow-up: 30.8 months [interquartile range, IQR: 12.9–57.4]). Univariable and multivariable Cox regression analyses were performed.

**Results:**

Secondary enucleation (SE) was performed in 13.5% of cases (n = 78) after the median of 20.0 months (IQR: 9.0–34.7) post-BBNP. The overall rate of local tumor recurrence (LR) in the cohort was 8.5% (n = 49) and was diagnosed at the median post-BBNP interval of 20.0 months (IQR: 15.6–35.2). Of the patients’ baseline characteristics, higher age (> 67 years, adjusted hazard ratio [aHR] = 1.80, *p =* 0.011), tumor thickness (> 8.5 mm, aHR = 2.20, *p =* 0.002), visual acuity (> 0.5 logMAR, aHR = 1.83, *p =* 0.009), and sclera dose (> 1000 Gy, aHR = 1.65, *p =* 0.034) were independently associated with the risk of SE. In turn, higher age (> 67 years, aHR = 1.93, *p =* 0.023), tumor thickness (> 8.5 mm, aHR = 2.02, *p =* 0.020), and visual acuity (> 0.5 logMAR, aHR = 2.27, *p =* 0.005) were independently related to LR.

**Conclusions:**

BBNP facilitates eye retention in 86.5% of patients with large uveal melanoma 2.5 years after treatment. Patients’ baseline, tumor and treatment characteristics were strongly associated with the risk of SE and LR after BBNP.

**Supplementary Information:**

The online version contains supplementary material available at 10.1186/s13014-025-02707-7.

## Introduction

Uveal melanoma (UM) arises from melanocytes in the uveal tract of eye and is the most frequent malignant intraocular tumor in adults [1]. The management of UM has evolved from enucleation, which has been a traditional therapy method before 1960s, to globe sparing and function preserving modalities like radiotherapy or combined therapies with surgical tumor resection, either via a transretinal or transscleral approach [2–4]. According to National Comprehensive Cancer Network guidelines for UM brachytherapy and particle beam radiotherapy are the primary therapy modalities for most cases, amenable to eye-conserving approaches [5]. While brachytherapy is more accessible and less expensive, proton beam irradiation (PBI) is limited in availability and comes with high operating costs, however, in terms of survival rates, both brachytherapy and PBI demonstrate similar efficacy overall [6].

Brachytherapy is currently considered the most commonly used therapy for UM, whereas ^125^Iodine (^125^I) and ^106^Ruthenium (^106^Ru) are the most commonly used radioisotopes [7]. Due to rapid dose fall-off, brachytherapy with beta emitter ^106^Ru is limited to tumors with thickness roughly up to approx. 7.0 mm depending on the size of the plaque [8–10]. Gamma-emitter ^125^I has a slower dose fall-off, which enables a sufficient irradiation of larger tumors, but at the same time means higher radiation exposure of neighboring radiosensitive structures [8]. The differences in radiation gradient between the two radionuclides formed the basis of the hypothesis that ^106^Ru may offer a considerable reduction of radiation exposure outside of the target volume [8, 11, 12]. Fittingly, significantly lower rate of post-radiation complications after brachytherapy with ^106^Ru than after ^125^I has been previously reported [11, 13].

Based on physical properties of above-mentioned radionuclides, bi-nuclide radioactive plaques (^106^Ru /.^125^I) aiming a local tumor control with reduced post-radiation toxicity in large UM with tumor thickness ≥ 7.0 mm, were developed at the Departments of Ophthalmology and Radiotherapy of the University Hospital Essen in 1997 [8]. The rationale behind this treatment approach with the combination of two radionuclides was an optimization of dose distribution, and therefore, an improvement of anatomical and functional outcome after brachytherapy of large UM. [8]

With this study, we want to share our 22-years’ experience of treatment of large UM by brachytherapy with bi-nuclide plaques (BBNP), with the special emphasis on eye retention and local tumor control rates.

## Methods and materials

### Study design and study population

This monocentric observational study was conducted using clinical data of patients with UM, treated with BBNP at the Departments of Ophthalmology and Radiotherapy of the University Hospital Essen between January 1999 and December 2020. The patients treated with two plaques as primary therapy (bi-nuclide and Ru) were excluded from the final analysis.

This trial was conducted in accordance with the Declaration of Helsinki and approval of the Institutional Ethics Committee (Medical faculty of the University Duisburg-Essen, registration number 20-9165-BO) was obtained. The study was registered in the German clinical trial registry (DRKS, Unique identifier: DRKS00019049, registration date 10.21.2019). The informed consent was obtained from all patients within the written treatment contract signed on admission. The post-treatment follow-up data were screened up to 06/2023.

### UM management

Each patient underwent a comprehensive ophthalmologic examination with measurement of best corrected visual acuity with decimal charts, slit lamp biomicroscopy, indirect ophthalmoscopy, ultrasonography or/and ultrasound biomicroscopy, fundus photography and optic coherence tomography. After clinical diagnosis of UM, systemic evaluation with liver ultrasonography/function tests and computer tomography of the chest was performed to exclude metastatic disease. Small tumors with thickness < 7 mm were treated with ^106^Ru plaques. The juxtapapillary and juxtamacular tumors were treated with PBI. Tumor thickness of ≥ 7 mm was an indication for BBNP in our center. At the initial introduction the prescribed minimum dose for the tumor apex was 120 Gy for tumors with a height of ≤ 8 mm and 100 Gy for tumors exceeding 8 mm in height. The scleral base received a dose of at least 700 Gy, but did not exceed 1500 Gy [14]. In 2001–2002, the prescribed dose was adjusted to 84 Gy for the apex in tumors ≤ 8 mm in height and 70 Gy for tumors > 8 mm. The duration of irradiation was calculated by radiation therapists and radiation physicists of the Department of Radiotherapy of the University Hospital Essen. After brachytherapy, all patients underwent regular follow-up at our outpatient service, initially every 3 months, with further extension of the follow-up intervals up to 6, 9 and 12 months.

Transpupillary thermotherapy (TTT) was performed with an 810-nm infrared laser and beam diameter of 2–3 mm. Prolonged exposure of laser beam (up to 1 min) induced localized heating, leading to tissue necrosis. TTT was applied as adjuvant therapy either as a “sandwich” therapy in cases with insufficient apex dose shortly after plaque brachytherapy or as a treatment to manage inadequate scar formation or local recurrences (LR) at the tumor margin. Any tumor growth 6 months after BBNP validated as an increase of tumor volume in ultrasonography or by photographic documentation was defined as LR. Depending on the extent of the recurrence, remaining visual acuity and the patient’ preferences, LR were treated either with a second brachytherapy, TTT, or a secondary enucleation (SE). We defined a lack of tumor regression with clinical evidence of active tumor 6 months after BBNP as insufficient tumor regression.

### Bi-nuclide plaque

**Fig. 1:**
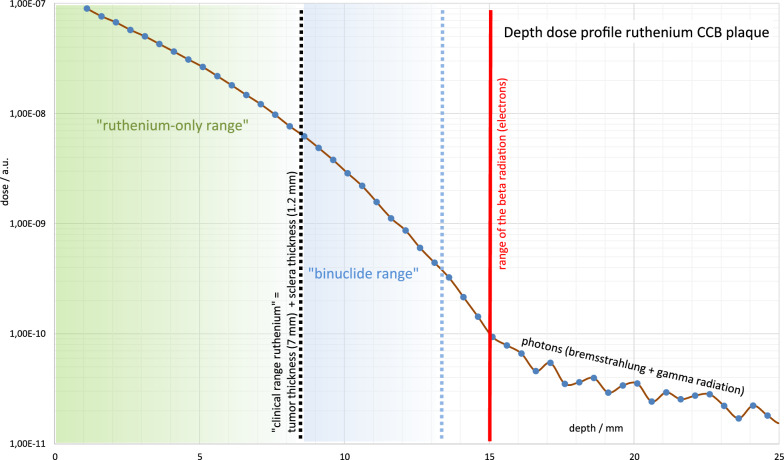
Depth dose distribution of ^125^I and a ^106^Ru eye plaques. ^106^Ru eye plaque profiles show a steep dose fall-off, limiting the therapeutic range to 7 mm tumor thickness (“ruthenium-only range”). In combination with ^125^I, tumors up to 12 mm can be irradiated (“bi-nuclide range”). Beyond the range of the emitted beta radiation, bremsstrahlung and gamma radiation can be observed

Bi-nuclide plaques (designed by W. Sauerwein and D. Flühs and manufactured by Schmuck Merath, Ulm, Germany) were crafted in the Department of Radiotherapy, University Hospital Essen, and consisted of a golden plaque with two fixation eyelets for suturing the plaque onto the eye, a regular 20 mm ^106^Ru plaque (CCB type manufactured by BEBIG, Berlin, Germany) which is embedded in a golden plaque and 8–12 symmetrically arranged ^125^I seeds (Amersham type 6711) in silicone inset.[8] Fig. 1. shows a dose distribution in bi-nuclide plaques. “^106^Ru only range” ends at a tumor height of approx. 7 mm (apex dose min. 100 Gy, scleral dose < approx. 1500 Gy). Including the scleral thickness of approx. 1.2 mm, this results in a dose depth of approx. 8.2 mm. Bi-nuclide plaques can be used up to about 12 mm tumor thickness (“bi-nuclide range”). The total apex dose comprised of ^106^Ru and ^125^I fractions, whereas ^106^Ru contribution depends on the tumor thickness, but also on the plaque itself: the depth dose curve of bi-nuclide plaque changes with the duration of use, as ^125^I decays faster (60 days) than ^106^Ru (374 days) [8]. The ^106^Ru fraction of the apex dose was < 10% in around 1/3 of all patients, > 10% and < 30% in half of the patients, > 30% in the remaining patients. In contrast to ^106^Ru, ^125^I consists exclusively of low-energy photons, demonstrates a significantly flatter curve and there is also no ‘kink in the curve’ as with ^106^Ru at a depth of 15 mm.

### Data management

The following data were extracted from the electronic medical records: patient age at diagnosis, sex, date of brachytherapy, the last documented follow-up, LR, SE and the underlying cause for enucleation (LR, insufficient tumor regression, total retinal detachment, phthisis bulbi, secondary glaucoma, painful eye, lack of tumor control, toxic tumor syndrome), largest tumor thickness and largest basal tumor diameter prior to the therapy (based on ultrasonography), ciliary body involvement, iris involvement, location of posterior tumor margin (peripapillary, posterior to equator, anterior to equator), extraocular tumor extension, intravitreal therapy with anti-vascular endothelial growth factor and/or triamcinolone, apex and sclera dose, radiation duration, occurrence of radiation induced scleral necrosis. The tumors with posterior tumor margin within 5 mm proximity to optic nerve were defined as peripapillary. Tumors were classified in categories according to the eighth edition of the American Joint Committee on Cancer tumor, node, metastasis (TNM) system [15].

Visual acuity (VA) was documented at first diagnosis of UM (pretreatment) with decimal scale chart at a distance of 5 m, recorded in declining order from 1.0 (20/20) to 0.05 (20/400). A VA worse than 0.02 (20/800) was recorded as counting fingers at 1 m, hand movement, light perception, and no light perception.

### Study endpoints and statistical analysis

The main endpoint of the study was to analyze the rate and the baseline risk factors related to SE and LR after BBNP. Assessing variables affecting the timing of SE and LR was the secondary endpoint of our study. Data analysis was performed using SPSS (version 25, SPSS Inc., IBM, Chicago, IL, USA). P-values of 0.05 or less were considered as significant. For descriptive statistics of continuous variables median values (with interquartile range [IQR]) was reported. Categorical variables were reported as absolute numbers (with percentages). For statistical analysis, VA data were converted to LogMAR units (logarithm of the Minimum Angle Resolution).

First, primary study endpoints were addressed using univariable Cox regression analysis with each of the recorded baseline parameters. The assumption of proportional hazards was verified using SPSS. The included variables fulfilled the assumption of proportional hazards. Prior to inclusion to the final regression analysis, continuous variables with significant associations were converted to dichotomous variables using the cutoffs upon the receiver operating characteristic (ROC) curves. Finally, the variables showing significance in univariable analysis were then included in multivariable Cox regression analysis, which revealed the independent predictors of the study endpoints. The impact of cumulative effect of the relevant predictors of SE and LR was analyzed using Kaplan–Meier-survival plots.

As the secondary study endpoint, the association between baseline characteristics and the timing of SE and LR was assessed in the sub-cohort of individuals who developed these complications during the follow-up. For dichotomous baseline parameters, the differences in the SE/LR timing were analyzed using the Student’s *t*-test for normally distributed data and the Mann–Whitney-U-test for non–normal distributed data. For the remaining parameters, the Pearson or Spearman correlation tests were applied, as appropriate.

## Results

We identified 594 patients with large UM (tumor thickness ≥ 7 mm) treated with BBNP at the Department of Ophthalmology and Radiotherapy of the University Hospital Essen between 01/1999 and 12/2020. Patients managed with two plaques as the primary therapy (bi-nuclide and Ru) (n = 18) were excluded from final analysis. Therefore, 576 patients were included in the final analysis. The major baseline patients, tumor and radiation characteristics are presented in Tabl﻿e 1. TTT was used as an adjuvant treatment in 25 (4.3%) patients, and in 3 (0.5%) patients for control of LR. The median post-treatment follow-up in the cohort was 30.8 months (IQR: 12.9–57.4).

**Table 1:** Major baseline and follow-up characteristics of the cohort

Parameter	Number of cases (%) or median value (IQR)
Age, years	65.4 (54.5–74.0)
Age, > 67 years	254 (44.1%)
Sex, female	278 (48.3%)
*TNM category*	
T2a	36 (6.3%)
T2b	42 (7.3%)
T3a	204 (35.4%)
T3b	226 (39.2%)
T3c	6 (1.0%)
T3d	17 (3.0%)
T4a	15 (2.6%)
T4b	28 (4.9%)
T4c	2 (0.3%)
Tumor thickness prior the therapy, mm	8.6 (7.9–9.6)
Tumor thickness > 8.5 mm	297 (51.7%)
Largest basal tumor diameter, mm	15.0 (13.3–16.4)
Posterior tumor margin*:	
Peripapillary	154 (36.0%)
Anterior to equator	140 (32.8%)
Posterior to equator	132 (31.0%)
Extraocular extension	28 (4.9%)
Ciliary body involvement	313 (54.3%)
Iris involvement	0 (0%)
Radiation induced scleral necrosis	68 (11.8%)
Intravitreal therapy with Anti-VEGF or triamcinolone	56 (9.7%)
Triamcinolone intravitreal during brachytherapy	193 (33%)
Transpupillary thermotherapy	25 (4.3%)
Apex dose, Gy	74.7 (70.6–87.0)
Sclera dose, Gy	939.4 (813.0–1105.7)
Sclera dose > 1000 Gy	223 (40.3%)
Radiation duration, hour	139.9 (100.8–184.8)
Time interval Brachytherapy–enucleation, months	20.0 (9.0–34.7)
Time interval Brachytherapy–local recurrence, months	20.0 (15.6–35.2)
Follow up duration, months	30.8 (12.9–57.4)

Abbreviations: IQR – interquartile range; WHO- World Health Organization; TNM-tumor, node, metastasis; Anti- VEGF- Anti–vascular endothelial growth factor; *- No available information in 150 patients

### Primary study endpoints: rate and risk factors for SE and LR after BBNP

SE was performed in 78 (13.5%) patients at the median post-BBNP interval of 20.0 months (IQR: 9.0–34.7). Life-table analysis demonstrated a five-year and ten-year enucleation-free rate of 78% and 75% respectively (s. supplementary table S1). The most common indications to SE were local tumor failure (n = 43, 55.1%), followed by insufficient tumor regression (n = 9, 11.5%), phthisis bulbi (n = 6, 7.7%), suspected tumor recurrence (without evidence of viable tumor cells in histopathological examination, n = 6, 7.7%), lack of the tumor control due to loss of visibility to the fundus (n = 5, 6.4%), retinal detachment (n = 3, 3.8%) and secondary glaucoma (n = 2, 2.6%). In three cases (3.8%), SE was performed due to painful blind eye. Finally, one patient (1.3%) with toxic tumor syndrome also underwent SE.

Insufficient tumor regression was diagnosed in 17 cases. Nine of these patients underwent SE, and additional brachytherapy with a ^106^Ru plaque was performed in 8 cases.

In 8.5% of patients (n = 49), a LR was diagnosed at the median post-BBNP interval of 20.0 months (IQR: 15.6–35.2). Life-table analysis demonstrated a five-year and ten-year LR-free rate of 85% and 79% respectively (s. supplementary table S2). The supplementary table S3 shows the management options of LR.

In the univariable Cox regression analysis, the following parameters were associated with SE (s. supplementary table S4): age > 67 (*p =* 0.014), tumor thickness > 8.5 mm (*p =* 0.001), visual acuity at diagnosis > 0.5 logMAR (*p =* 0.018), and sclera dose > 1000 Gy (*p =* 0.011). In the final multivariable Cox regression analysis (Table 2), patients’ age > 67 years (adjusted hazard ratio [aHR] = 1.80, *p =* 0.011), tumor thickness (> 8.5 mm, aHR = 2.20, *p =* 0.002), visual acuity (> 0.5 logMAR, aHR = 1.83, *p =* 0.009), and sclera dose (> 1000 Gy, aHR = 1.65, *p =* 0.034) were independently associated with the risk of SE.

**Table 2:** Multivariable Cox regression analysis of predictors for secondary enucleation after brachytherapy with bi-nuclide plagues of large uveal melanoma (tumor thickness ≥ 7.0 mm)

Parameter	aHR (95% CI)	*p*-value
Age, > 67 years	1.80 (1.15–2.81)	**0.011**
Tumor thickness, > 8.5 mm	2.20 (1.35–3.58)	**0.002**
Visual acuity at diagnosis, > 0.5 logMAR	1.83 (1.16–2.87)	**0.009**
Sclera dose, > 1000 Gy	1.65 (1.04–2.61)	**0.034**

Abbreviations: aHR-adjusted hazard ratio; CI- Confidence interval

Bold values indicate statistically significant results (*p*<0.05)

In the univariable analysis of LR predictors (s. supplementary table S5), age > 67 (*p =* 0.026), tumor thickness > 8.5 mm (*p =* 0.032) and visual acuity at diagnosis > 0.5 logMAR (*p =* 0.010) showed significant results, and were also confirmed as independent predictors in the final multivariable Cox regression analysis (Table 3): patients’ age > 67 years (aHR = 1.93, *p =* 0.023), tumor thickness (> 8.5 mm, aHR = 2.02, *p =* 0.020), and visual acuity (> 0.5 logMAR, aHR = 2.27, *p =* 0.005).

**Table 3:** Multivariable Cox regression analysis of the predictors for local recurrence after brachytherapy with bi-nuclide plagues of large uveal melanoma (tumor thickness ≥ 7.0 mm)

Parameter	aHR (95% CI)	p-value
Age, > 67 years	1.93 (1.10–3.40)	**0.023**
Tumor thickness, > 8.5 mm	2.02 (1.12–3.65)	**0.020**
Visual acuity at diagnosis, > 0.5 logMAR	2.27 (1.29–3.99)	**0.005**

Abbreviations: aHR-adjusted hazard ratio; CI- Confidence interval

Bold values indicate statistically significant results (*p*<0.05)

**Fig. 2:**
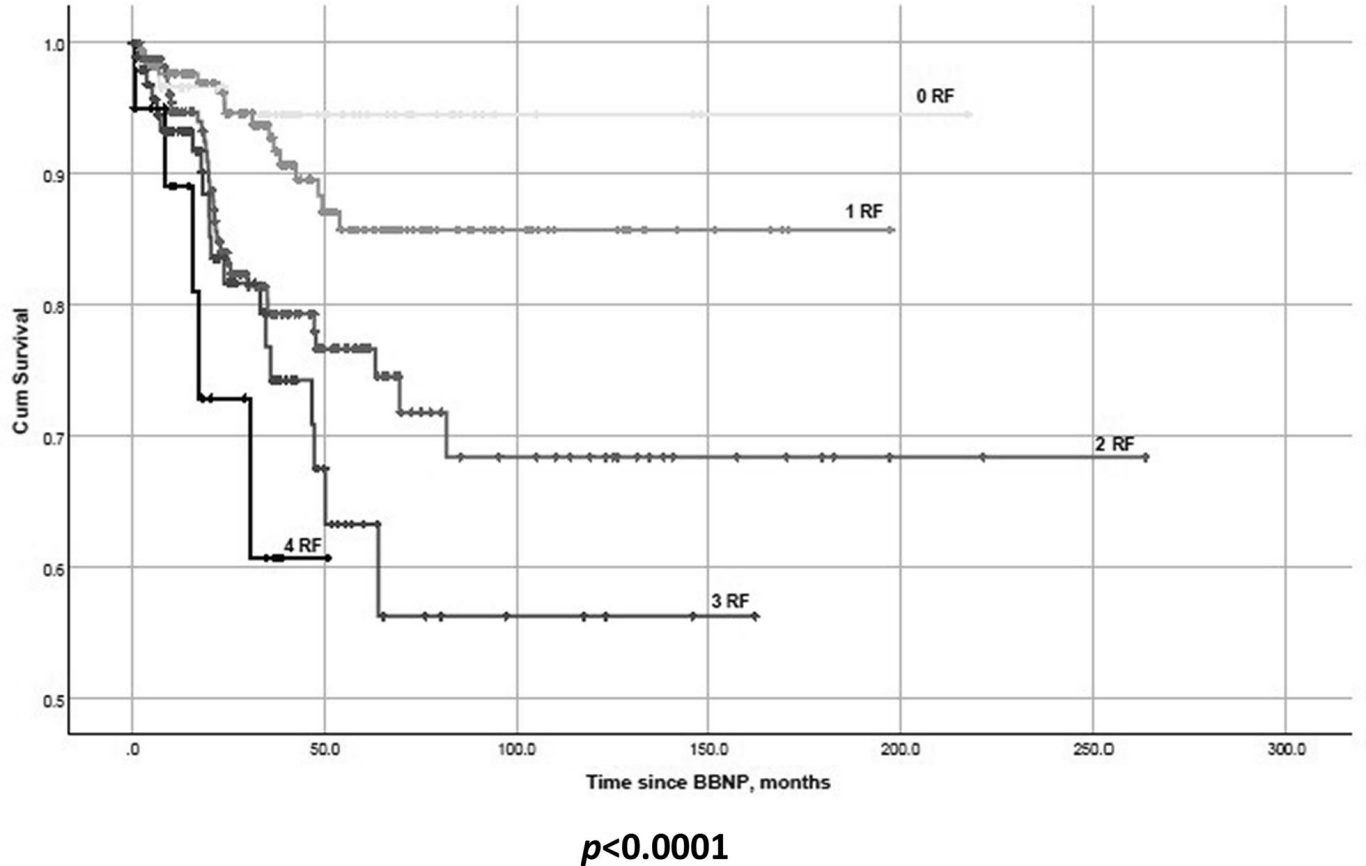
Kaplan–Meier-survival plot showing the need for secondary enucleation in the post-BBNP follow-up depending on the number of independent risk factors (patients’ age > 67 years, tumor thickness > 8.5 mm, visual acuity > 0.5 logMAR, and sclera dose > 1000 Gy) present at treatment begin. **Abbreviations**: RF–risk factors

**Fig. 3:**
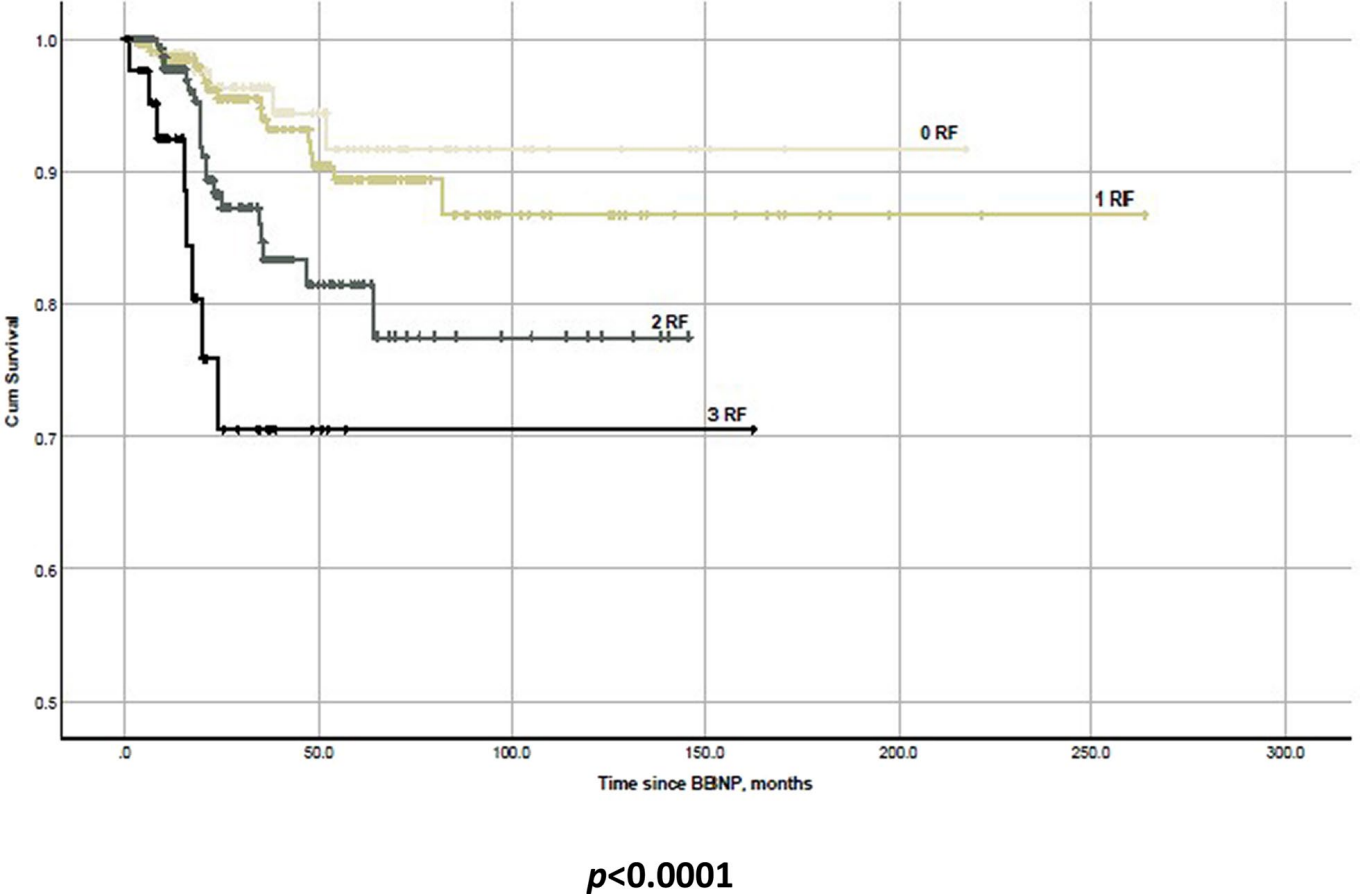
Kaplan–Meier-survival plot showing local tumor recurrence in the cohort depending on the number of present risk factors (patients’ age > 67 years, tumor thickness > 8.5 mm, visual acuity > 0.5 logMAR) at treatment begin. **Abbreviations**: RF–risk factors

Using the Kaplan–Meier survival analysis, we showed the cumulative effect of the significant predictors of SE (Fig. 2) and occurrence of LR (Fig. 3).

### Secondary study endpoints: the factors impacting the timing of SE and LR after BBNP

**Fig. 4:**
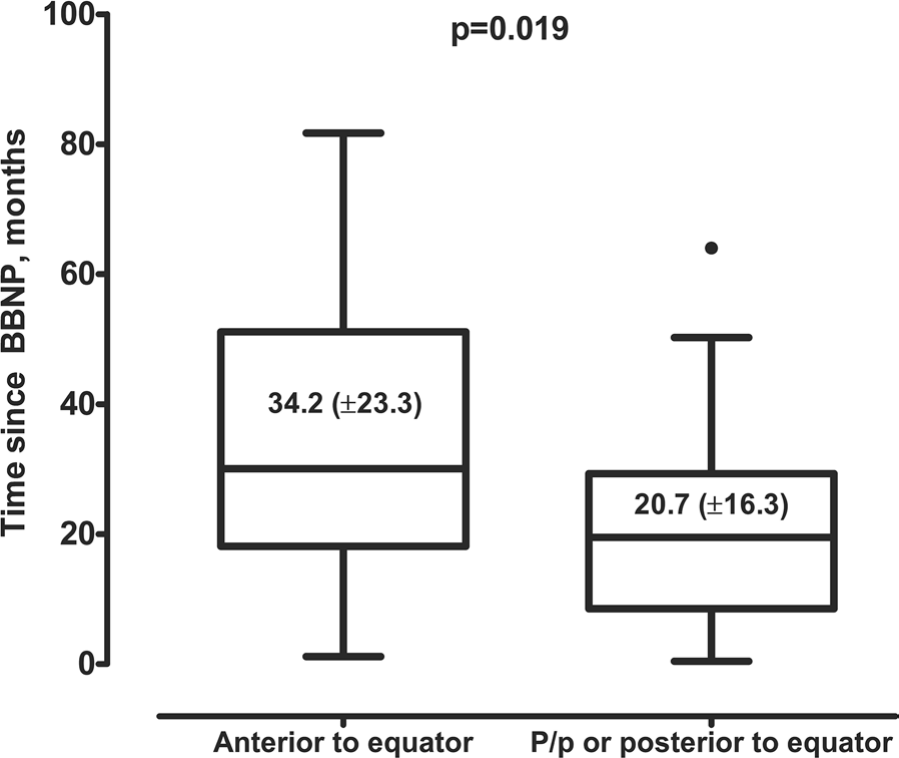
Box plots showing the timing of secondary enucleation after brachytherapy with bi-nuclide plagues of large uveal melanoma (tumor thickness ≥ 7.0 mm) depending on the location of the posterior tumor margin: anterior to equator vs peripapillary (P/p) or posterior to equator

We have analyzed the impact of the baseline variables on the timing of SE and LR (s. supplementary table S6). As visualized in Fig. 4, the location of the posterior tumor margin anterior to equator significantly prolonged the time until SE (mean: 34.2 ± 23.3 vs. 20.7 ± 16.3 months, r = 0.325/*p =* 0.019). Other parameters showed no impact on the timing of SE and LR.

## Discussion

In this large retrospective single-center study, BBNP was confirmed as an effective therapy option for UM with thickness exceeding 7 mm. Of 576 eyes treated with BBNP, 86.5% of affected eyes were salvaged after median follow up of 30.8 months. LR was diagnosed in 8.5% of cases and was treated with SE in almost 88% of cases.

Brachytherapy is the most common therapy modality for UM, which is widely accessible and less expensive than other forms of radiotherapy [6]. In the largest study evaluating contemporary treatment trends for UM, an increased rate of episcleral brachytherapy in the management of thick UM was reported [16]. Alongside with standardized set of episcleral plaques made of gold alloy with silastic inserts for ^125^I seeds used in the Collaborative Ocular Melanoma Study (COMS), different episcleral plaques have been developed in ocular oncology with the aim to minimize radiation doses for critical ocular structures and decrease the rates of radiation toxicity while maintaining sufficient tumor irradiation and margin coverage [17–24]. The concepts have been used to achieve an optimization of dose distribution in certain clinical scenarios consisted of use of a new seed model, plaque design and shapes adjusted to various tumor shapes, use of treatment planning system as well as using dual radioactive ^125^I source strengths [17–24].

The strategy for plaque optimization in bi- nuclide plaques is based on the physical characteristics of ^125^I and ^106^Ru. ^125^I is gamma -emitter with photon energies 25–35 keV and dose fall-off factor of 2 per 4–5 mm water, whereas beta- emitter ^106^Ru with a maximum energy 3.5 MeV has the fall-off factor of 2 per 2–2.5 mm water [8]. A combination of low-energy ^125^I and high energy ^106^Ru facilitates deeper radiation penetration with simultaneous significant decrease of the dose delivered to the adjacent radiosensitive structures. The latter is of crucial importance since radiation induced complications are dose associated and can even be a reason for SE [25].

^125^I and ^106^Ru are the most commonly used radioisotopes for brachytherapy of ocular tumors [7]. Due to limited depth of penetration, ^106^Ru-plaques are generally considered to be insufficient for tumors with thickness > 7 mm, which means higher risk for LR and, subsequently, the need for SE [26]. In the first report of large UM greater than 8 mm treated with ^106^Ru brachytherapy, SE was performed in approximately one third of the patients [27]. In one of the publications reporting the outcome after brachytherapy of large UM, ^106^Ru radionuclide was estimated as an independent risk factor for SE [28]. Interestingly, in a study comparing the globe preserving rate after ^106^Ru brachytherapy of large (> 7 mm) and medium-sized (< 7 mm) UM, there was no significant difference (90.7% vs. 92.6%, *p =* 0.614) between the two groups [29].

Brualla et al. published a guidance on the largest tumor height value that can be treated with a ^106^Ru plaque based on dosimetric results obtained from the simulations, taking into account the centric or eccentric placement of the plaque [30]. They estimated that ^106^Ru brachytherapy of tumors exceeding 6.5 mm in thickness and receiving the minimum prescribed dose of 100 Gy leads to sight-threatening doses being delivered to the neighbor structures. A combination of ^106^Ru with ^125^I can facilitate sufficient irradiation of larger tumors with concurrent reducing irradiation of structures besides the tumor volume.

In our study, 78 of 576 (13.5%) patients underwent SE at the median post-BBNP interval of 20.0 months. We found that patients’ age > 67 years, tumor thickness > 8.5 mm, visual acuity at diagnosis > 0.5 logMAR, and sclera dose > 1000 Gy were predictors for SE. While some of this parameters such as greater tumor thickness [28, 31, 32] and poorer baseline visual acuity in the affected eye [31, 33, 34] have been already reported as predictors of SE after episcleral brachytherapy, we identified also a new risk factor, namely the applied scleral dose. Higher SE rate in these patients can be explained with higher rate of radiation toxicity. On the other hand, tumor thickness and scleral dose are biologically related to each other: achieving a certain apex dose in thicker tumors inevitably means a higher applied scleral dose, which may serve as a surrogate for tumor thickness.

Regarding the patients’ age, older age has been known rather as LR predictor than of SE [31].

The 5-years risk for SE in our cohort was estimated at 22%, closely aligning with the 24% reported after ^125^I brachytherapy of UM with tumor thickness > 8 mm and the 22.6% observed following PBI for large UM [28, 35]. We have estimated the 10-year risk for SE to be 25%, which remains stable in the further course and is considerably lower than 34% previously reported by Shields et al.[28] In comparison, the 10-year risk of SE after PBI for large UM was estimated at 29.6% [35]. Our results demonstrated that the majority of SE of the eyes treated with BBNP were performed in the first 5 years (supplementary table S1). Generally, SE is indicated in case of LR, insufficient tumor regression or intolerable radiation induced complications, such as neovascular glaucoma or scleral necrosis [36–38]. Of note, the proportion of patients secondary enucleated due to radiation induced complications was considerably lower in our cohort than in previously reported study[39] (25.6% vs. 72%). One recent publication dealing with retrospective analysis of SE for UM after plaque radiotherapy revealed neovascular glaucoma (39%) as the major cause of SE [38]. In our cohort, 2 (2.6%) patients were enucleated due to secondary glaucoma. Taking into consideration that a higher radiation dose to adjacent tissue has been already reported as a risk factor for the development of secondary glaucoma [40], we hypothesize that radiation exposure of neighbor structures and radiation induced complications in our cohort was lower. With BBNP representing a synergy of two radionuclides, we have achieved one of the most important goals in radiotherapy: minimizing radiation-induced complications through reduction of radiation exposure outside of the target volume, while maintaining a high rate of local tumor control.

Apart of radiation toxicity, maintenance of the local tumor control is of crucial importance, since there is a significant difference in survival after treatment of UM between groups of patients with and without LR [41]. The rate of reported LR after brachytherapy for large UM with different radionuclides ranges from 5 to 24% [27–29, 39]. In a meta-analysis on LR rate after globe preserving therapy, the maintained risk for developing of LR was 9.6% both after ^125^I or ^106^Ru brachytherapy [42].

We have diagnosed the LR in 8.5% of patients at the median 20.0 months after BBNP. Our results are in line with the observations from previous publications, reporting LR rates, ranging from 6 to 9.6% [39, 41, 42]. According to our results, the 5-years risk to develop a LR after BBNP was 15%, and 10-years risk was 21%, which remained stable in further course (s. supplementary table S2). The long-term outcomes in large UM have also been addressed by Papakostas et al., who reported slightly better results, with a 5-year risk of LR after PBI at 7.8%, increasing to 12.5% after 10 years [35].

In our cohort, patients’ age > 67 years, tumor thickness > 8.5 mm and visual acuity at diagnosis > 0.5 logMAR were confirmed as independent predictors of LR. Older age and greater tumor thickness alongside with proximity of the tumor to the foveal avascular zone have been already described as independent risk factors for LR in COMS Report Nr 19, exploring local treatment failure in the first five years after ^125^I brachytherapy [31]. Consistent with our results, Barker et al. also reported an association of a low visual acuity in the affected eye with LR [34]. Another reported risk factors predisposing to LR after brachytherapy are the largest basal diameter, the juxtapapillary location, the involvement of the ciliary body, brachytherapy with ^106^Ru and the use of adjuvant TTT [28, 41, 43, 44].

The retrospective nature of our study is certainly a study limitation. Lower accuracy and incompleteness of the recoded data may affect the study's validity. Although the treatment decisions in the analyzed cohort, particularly regarding SE, were met in accordance with the institutional standard operating procedures, the absence of a strict study protocol regulating the treatment process leaves certain space for individual preferences of treating ophthalmologists. Moreover, patients are also known to impact the treatment decisions. These circumstances lead to selection bias which cannot be fully ruled out during the analysis. Nevertheless, our study is based on a large representative cohort with the assessment of independent associations between a broad range of potential predictors and the study endpoints.

## Conclusions

In this first study evaluating the anatomical outcome after BBNP in UM with tumor thickness ≥ 7.0 mm, BBNP provided an effective therapy with eye preservation in 86.5% and local tumor control in 91.5% of cases 2.5 years after the initial treatment. Patients’ age > 67 years, tumor thickness > 8.5 mm and visual acuity at diagnosis > 0.5 logMAR were identified as independent risk factors for both LR and SE. In addition, scleral dose > 1000 Gy was also associated for the risk of SE. Further evaluation and optimization of treatment regimes in patients with large UM are essential for achieving better anatomic and functional results.

## Supplementary Information

Supplementary Material 1

Supplementary Material 2

Supplementary Material 3

Supplementary Material 4

Supplementary Material 5

Supplementary Material 6

## Data Availability

No datasets were generated or analysed during the current study.

## Declarations

### Clinical trial registration

The Study was registered in the German clinical trial registry (DRKS, Unique identifier: DRKS00019049, registration date 10.21.2019).
