# Supplementary material for: Anatomical outcome after brachytherapy with bi-nuclide (Ru-106/Iodine-125) plaques in large uveal melanomas
Source: Radiat Oncol. 2025 Jul 31;20:119. doi: 10.1186/s13014-025-02707-7 (PMC12315396; doi:10.1186/s13014-025-02707-7)
Supplement: Supplementary file 1 — Supplementary Material 1 [file 13014_2025_2707_MOESM1_ESM.docx]

**Table S1**: Life table demonstrating the probability of enucleation after brachytherapy with bi-nuclide plagues of large uveal melanoma (tumor thickness ≥7 mm)

| **Interval Start Time**  **(months)** | **Number Entering Interval** | **Number Withdrawing during Interval** | **Number of Terminal Events** | **Proportion Surviving** | **Cumulative Proportion Surviving at End of Interval** |
| --- | --- | --- | --- | --- | --- |
| 0 | 576 | 118 | 20 | 0.96 | 0.96 |
| 12 | 438 | 76 | 22 | 0.95 | 0.91 |
| 24 | 340 | 69 | 14 | 0.95 | 0.87 |
| 36 | 257 | 60 | 10 | 0.96 | 0.83 |
| 48 | 187 | 46 | 7 | 0.96 | 0.79 |
| 60 | 134 | 41 | 2 | 0.98 | 0.78 |
| 72 | 91 | 24 | 2 | 0.97 | 0.76 |
| 84 | 65 | 16 | 1 | 0.98 | 0.75 |
| 96 | 48 | 9 | 0 | 1.00 | 0.75 |
| 108 | 39 | 7 | 0 | 1.00 | 0.75 |
| 120 | 32 | 32 | 0 | 1.00 | 0.75 |
| 132 | 23 | 6 | 0 | 1.00 | 0.75 |
| 144 | 17 | 4 | 0 | 1.00 | 0.75 |
| 156 | 13 | 3 | 0 | 1.00 | 0.75 |
| 168 | 10 | 4 | 0 | 1.00 | 0.75 |
| 180 | 6 | 1 | 0 | 1.00 | 0.75 |
| 192 | 5 | 2 | 0 | 1.00 | 0.75 |
| 204 | 3 | 0 | 0 | 1.00 | 0.75 |
| 216 | 3 | 2 | 0 | 1.00 | 0.75 |
| 228 | 1 | 0 | 0 | 1.00 | 0.75 |
| 240 | 1 | 0 | 0 | 1.00 | 0.75 |
| 252 | 1 | 1 | 0 | 1.00 | 0.75 |
