# Supplementary material for: Anatomical outcome after brachytherapy with bi-nuclide (Ru-106/Iodine-125) plaques in large uveal melanomas
Source: Radiat Oncol. 2025 Jul 31;20:119. doi: 10.1186/s13014-025-02707-7 (PMC12315396; doi:10.1186/s13014-025-02707-7)
Supplement: Supplementary file 3 — Supplementary Material 3 [file 13014_2025_2707_MOESM3_ESM.docx]

**Table S3:** Management of local recurrence after brachytherapy with bi-nuclide plagues of large uveal melanoma (tumor thickness ≥7 mm)

| **Therapy option** | **Number of cases (%)** |
| --- | --- |
| Re-brachytherapy* | 5 (10.2%) |
| TTT | 2 (4.1%) |
| Re-brachytherapy + TTT | 1 (2.0%) |
| SE | 43 (87.8%) * |

Abbreviations: TTT- transpupillary thermotherapy; SE-Secondary enucleation; * - Two patents managed with re-brachytherapy underwent SE due to repeated recurrence
