# Supplementary material for: Anatomical outcome after brachytherapy with bi-nuclide (Ru-106/Iodine-125) plaques in large uveal melanomas
Source: Radiat Oncol. 2025 Jul 31;20:119. doi: 10.1186/s13014-025-02707-7 (PMC12315396; doi:10.1186/s13014-025-02707-7)
Supplement: Supplementary file 4 — Supplementary Material 4 [file 13014_2025_2707_MOESM4_ESM.docx]

**Table S4**: Univariable Cox regression analysis of the predictors of secondary enucleation after brachytherapy with bi-nuclide plagues of large uveal melanoma (tumor thickness ≥ 7.0 mm)

| **Parameter** | **HR (95% CI)** | **p-value** |
| --- | --- | --- |
| Age, (per year increase) | 1.02 (1.01-1.04) | 0.007 |
| Age, >67 years | 1.76 (1.12-2.74) | **0.014** |
| Sex, female | 0.96 (0.62-1.50) | 0.868 |
| TNM, T4 grade vs T2 or T3 | 1.12 (0.49-2.58) | 0.785 |
| Tumor thickness prior the therapy, mm | 1.50 (1.29-1.74) | < 0.0001 |
| Tumor thickness >8.5mm | 2.28 (1.41-3.69) | **0.001** |
| Largest basal tumor diameter, mm | 1.01 (0.92-1.12) | 0.778 |
| Posterior tumor margin:  Peripapillary vs any other location  Anterior to equator vs any other location  Posterior to equator vs any other location | 0.94 (0.53-1.65)  0.90 (0.51-1.60)  1.20 (0.68-2.11) | 0.812  0.720  0.534 |
| Radiation induced scleral necrosis | 1.33 (0.75-2.38) | 0.332 |
| Extraocular extension | 0.94 (0.30- 3.00) | 0.923 |
| Ciliary body involvement | 1.00 (0.64-1.57) | 0.990 |
| Visual acuity at diagnosis, per logMAR | 1.70 (1.20-2.40) | **0.003** |
| Visual acuity at diagnosis >0.5 logMAR | 1.72 (1.10-2.70) | **0.018** |
| Adjuvant transpupillary thermotherapy | 0.46 (0.11-1.85) | 0.271 |
| Apex dose, per Gy | 0.99 (0.97-1.00) | 0.143 |
| Sclera dose, per Gy | 1.00 (1.00-1.00) | **0.005** |
| Sclera dose >1000 Gy | 1.79 (1.14-2.81) | **0.011** |
| Radiation duration, per Gy/h | 1.00 (0.99-1.00) | 0.542 |

**Abbreviations:** HR- Hazard ratio; CI- Confidence interval; TNM-tumor, node, metastasis.
