# Supplementary material for: Anatomical outcome after brachytherapy with bi-nuclide (Ru-106/Iodine-125) plaques in large uveal melanomas
Source: Radiat Oncol. 2025 Jul 31;20:119. doi: 10.1186/s13014-025-02707-7 (PMC12315396; doi:10.1186/s13014-025-02707-7)
Supplement: Supplementary file 5 — Supplementary Material 5 [file 13014_2025_2707_MOESM5_ESM.docx]

**Table S5:** Univariable Cox regression analysis of the predictors of local recurrence after brachytherapy with bi-nuclide plagues of large uveal melanoma (tumor thickness ≥ 7.0 mm)

| **Parameter** | **HR (95% CI)** | **p-value** |
| --- | --- | --- |
| Age, per year increase | 1.02 (1.00-1.05) | **0.034** |
| Age, >67 years | 1.90 (1.08-3.33) | **0.026** |
| Sex, female | 0.82 (0.46-1.43) | 0.478 |
| TNM, T4 grade vs T2 or T3 | 1.13 (0.40-3.15) | 0.810 |
| Tumor thickness prior the therapy, mm | 1.27 (1.03-1.56) | **0.023** |
| Tumor thickness >8.5mm | 1.90 (1.06-3.42) | **0.032** |
| Largest basal tumor diameter, mm | 1.04 (0.92-1.18) | 0.501 |
| Posterior Tumor margin:  Perypapillary vs any other location  Anterior to equator vs any other location  Posterior to equator vs any other location | 0.72 (0.34-1.49)  1.16 (0.58-2.31)  1.20 (0.60-2.40) | 0.372  0.667  0.616 |
| Radiation induced scleral necrosis | 1.40 (0.68-2.90) | 0.354 |
| Extraocular extension | 2.16 (0.77-6.00) | 0.141 |
| Ciliary body involvement | 1.00 (0.57-1.76) | 0.998 |
| Visual acuity at diagnosis, per logMAR | 1.68 (1.08-2.60) | **0.020** |
| Visual acuity at diagnosis >0.5 logMAR | 2.09 (1.19-3.67) | **0.010** |
| Adjuvant transpupillary thermotherapy | 0.35 (0.05-2.56) | 0.303 |
| Apex dose, per Gy | 0.98 (0.96-1.00) | 0.063 |
| Sclera dose, per Gy | 1.00 (1.00-1.00) | 0.287 |
| Sclera dose >1000 Gy | 1.50 (0.85-2.67) | 0.163 |
| Radiation duration, per Gy/h | 1.00 (0.99-1.00) | 0.454 |

**Abbreviations:** HR- Hazard ratio; CI- Confidence interval; TNM-tumor, node, metastasis.
