# Supplementary material for: Anatomical outcome after brachytherapy with bi-nuclide (Ru-106/Iodine-125) plaques in large uveal melanomas
Source: Radiat Oncol. 2025 Jul 31;20:119. doi: 10.1186/s13014-025-02707-7 (PMC12315396; doi:10.1186/s13014-025-02707-7)
Supplement: Supplementary file 6 — Supplementary Material 6 [file 13014_2025_2707_MOESM6_ESM.docx]

**Table S6:** Impact of basic variables on the timing of secondary enucleation (A) and local recurrence (B) after brachytherapy with bi-nuclide plagues of large uveal melanoma (tumor thickness ≥ 7.0 mm).

A:

| **Parameter** | **mean, (±SD)**  **with parameter** | **mean, (±SD)**  **without parameter** | **p-value** |
| --- | --- | --- | --- |
| Age >67 years | 21.5 (±17,0**)** | 25.8 (±18.6) | 0.197 |
| Sex, female | 23.4 (±17.0) | 23.8 (±18.7) | 0.976 |
| TNM, T4 vs T2 or T3 | 20.0 (**±**17.6) | 23.9 (±17.9) | 0.414 |
| Tumor thickness > 8.5mm | 23.4 (±16.9) | 24.2 (± 20.0) | 0.865 |
| Posterior Tumor margin: |  |  |  |
| PTM: Perypapillary vs any other location | 18.6 (±16.3) | 28.6 (±20.7) | 0.083 |
| PTM: Anterior to equator vs any other location | 34.2 (±23.3) | 20.7 (±16.3) | **0.039** |
| PTM: Posterior to equator vs any other location | 22.9 (±16.6) | 26.2 (±21.2) | 0.755 |
| Radiation induced scleral necrosis | 30.4 (±20.1) | 22.1 (±17.1) | 0.109 |
| Ciliary body involvement | 25.9 (±20.0) | 20.8 (±14.7) | 0.393 |
| Extraocular extension | 20.9 (±1.8) | 23.7 (±18.2) | 0.931 |
| Visual acuity at diagnosis >0.5 logMAR | 20.4 (±16.3) | 25.9 (±18.7) | 0.155 |
| Adjuvant transpupillary thermotherapy | 25.4 (±7.7) | 23.6 (±18.0) | 0.657 |
| Apex dose >75 Gy | 22.7 (±17.3) | 25.1 (±18.6) | 0.689 |
| Sclera dose >1000 Gy | 26.2 (±20.3) | 21.5 (±14.7) | 0.556 |

**Abbreviations:** PTM- posterior tumor margin: TNM-tumor, node, metastasis, SD-standard deviation

B:

| **Parameter** | **mean, (±SD)**  **with parameter** | **mean, (±SD)**  **without parameter** | **p-value** |
| --- | --- | --- | --- |
| Age >67 years | 23.2 (±17.0**)** | 27.3 (±16.4) | 0.276 |
| Sex, female | 28.0 (±16.5) | 22.9 (±16.7) | 0.165 |
| TNM, T4 vs T2 or T3 | 24.9 (**±**19.9) | 25.2 (±16.6) | 0.738 |
| Tumor thickness > 8.5mm | 22.7 (±14.3) | 29.9 (± 20.1) | 0.144 |
| Posterior Tumor margin: |  |  |  |
| PTM: Perypapillary vs any other location | 27.1 (±14.7) | 24.4 (±19.4) | 0.460 |
| PTM: Anterior to equator vs any other location | 28.5 (±22.6) | 23.3 (±14.9) | 0.724 |
| PTM: Posterior to equator vs any other location | 22.9 (±16.6) | 20.0 (±14.9) | 0.278 |
| Radiation induced scleral necrosis | 26.3 (±19.4) | 25.0 (±16.3) | 0.949 |
| Ciliary body involvement | 28.5 (±19.5) | 21.2 (±11.6) | 0.387 |
| Extraocular extension | 16.7 (±6.9) | 25.9 (±17.1) | 0.406 |
| Visual acuity at diagnosis >0.5 logMAR | 21.3 (±14.5) | 28.7 (±18.0) | 0.107 |
| Adjuvant transpupillary thermotherapy | 20.4 (*) | 25.3 (±16.8) | 0.980 |
| Apex dose >75 Gy | 27.3 (±15.8) | 25.3 (±17.4) | 0.504 |
| Sclera dose >1000 Gy | 29.1 (±19.1) | 22.9 (±13.8) | 0.469 |

**Abbreviations:** PTM- posterior tumor margin, TNM-tumor, node, metastasis, SD-standard deviation, *-only one patient in this subgroup.
